# Prediction of Epidemics Trend of COVID-19 in Bangladesh

**DOI:** 10.3389/fpubh.2020.559437

**Published:** 2020-11-30

**Authors:** Raguib Hassan, Abu Sayem Dosar, Joytu Kumar Mondol, Tahmid Hassan Khan, Abdullah Al Noman, Mirajus Salehin Sayem, Moinul Hasan, Nasrin Sultana Juyena

**Affiliations:** ^1^Faculty of Agriculture, Bangladesh Agricultural University, Mymensingh, Bangladesh; ^2^Faculty of Veterinary Science, Bangladesh Agricultural University, Mymensingh, Bangladesh; ^3^Faculty of Agricultural Engineering and Technology, Bangladesh Agricultural University, Mymensingh, Bangladesh; ^4^Department of Surgery and Obstetrics, Faculty of Veterinary Science, Bangladesh Agricultural University, Mymensingh, Bangladesh

**Keywords:** coronavirus, mathematical prediction model, pandemic (COVID-19), susceptible-infectious-recovered (SIR) model, graphical trend analysis

## Abstract

**Background:** Amid a critical and emergent situation like the coronavirus disease (COVID-19) pandemic related to extreme health and economic repercussions, we used and presented the mathematical modeling like susceptible–infectious–recovered (SIR) to have a numerical demonstration that can shed light to decide the fate of the scourge in Bangladesh. To describe the idea about the factors influencing the outbreak data, we presented the current situation of the COVID-19 outbreak with graphical trends.

**Methods:** Primary data were collected and analyzed by using a pre-created Google Survey form having a pre-set questionnaire on the social distancing status of different districts. Secondary data on the total and the daily number of laboratory tests, confirmed positive cases, and death cases were extracted from the publicly available sources to make predictions. We estimated the basic reproduction number (*R*_◦_) based on the SIR mathematical model and predicted the probable fate of this pandemic in Bangladesh.

**Results:** Quarantine situations in different regions of Bangladesh were evaluated and presented. We also provided tentative forecasts until 31 May 2020 and found that the predicted curve followed the actual curve approximately. Estimated *R*_◦_-values (6.924) indicated that infection rate would be greater than the recovery rate. Furthermore, by calibrating the parameters of the SIR model to fit the reported data, we assume the ultimate ending of the pandemic in Bangladesh by December 2022.

**Conclusion:** We hope that the results of our analysis could contribute to the elucidation of critical aspects of this outbreak and help the concerned authority toward decision making.

## Introduction

The whole world is literally shut down due to a minute RNA virus. Bangladesh is no exception in getting the coronavirus disease (COVID-19) as an imminent threat. The novel coronavirus SARS-CoV-2 from the Coronaviridae group causing COVID-19 was first identified in Wuhan province, China. After the spread in China, it continued to infect people from every corner of the globe, leaving almost two million people infected and hundreds of thousands of people dead (until 17 April 2020). This disease is transmitted by inhalation or contact with infected droplets or fomites, and the incubation period may range from 2 to 14 days (1). Due to its dangerously fast spread nature, WHO had to announce it a global pandemic on 11 March 2020 (2).

In Bangladesh, the first COVID-19 case was reported on 8 March 2020 [REUTERS (3)]. Two men (from Narayangonj district) and one woman (from Madaripur district) were tested positive for COVID-19. Of them, two men were Italy returnees, and the woman was a family member of one of these two. After a few days, the number started to increase in a geometrical pattern. Right then, it was only localized and individual spread. Since 26 March 2020, Bangladesh has been under a nationwide lockdown, with limited movements of emergency service personnel, to curb the spread of the new virus as a control measure along with other initiatives taken by the Government of the People's Republic of Bangladesh, as presented in Table 1, to prevent and control the pandemic spread. However, community spread/infection occurred over time. Bangladesh seems to be at a greater risk because of a high population density and limited infrastructure in healthcare systems to cater to very large demands (4). We aimed to present the future situation of the COVID-19 outbreak in Bangladesh with graphical trends considering the current situation. This study also aimed to deliver an overview of the susceptible–infectious–recovered (SIR) model and the outcome of our simulation by using the dataset of COVID-19. The SIR model is a kind of compartmental model describing the dynamics of infectious disease (5), and the SIR model is believed to have better abilities for long-term forecasts than the regression model. In the SIR model, an exact analytical solution is reproduced from the numerical solution (6). It is not a broad-term study, and many factors responsible for epidemiological differences (patient's age, gender, immunity status, viral virulence, etc.) had to be ignored due to the unavailability of data and lack of opportunity to gather data from field level in a short time. This is a small-scale initiative of visualizing the current situation and predicting or forecasting COVID-19 spread in Bangladesh.

**Table 1:** Measures taken by the Government of the People's Republic of Bangladesh.

**Date**	**Major activities against corona**
21 January 2020	Thermal screening for passengers in all international airports, land ports, and sea ports receiving passengers from the COVID-19 affected countries. Quarantine of passengers with fevers, coughs, breathing difficulties, and sore throats.
10 March 2020	Quarantine of individuals without symptoms (with history of transport abroad and individuals having history of contact of foreign returnees or infected individuals Isolation of individuals (imported or local) with positive symptoms
17 March 2020	A ban on incoming flights from the European destinations.
26 March 2020	Closure of all public and private offices
31 March 2020	A ban on people's travel via water, rail, and domestic air routes and public transports.
5 April 2020	Announcement of a stimulus package amounting to some US$8bn to restrict the mass flow of garment workers.

## Methods

### Data

We collected primary data using a pre-created Google Survey form having a pre-set questionnaire during 8–14 April 2020, which was shared in some public university social media forum where people from different regions willingly filled up the survey form. In total, 733 people from different regions took part in that survey to study the quarantine situations in different regions of Bangladesh. To avoid complications, only qualitative primary data were collected, and hence, the authenticity of the collected data completely depends on the participants of the survey. Primary data related to quarantine status were updated until 10 April 2020. Different terminologies presented in Table 2 were used to define the mode of transmission during this observation. Updated secondary data from the Institute of Epidemiology, Disease Control, and Research website (3) and the World Health Organization (8) until 14 April 2020 were used in graphs and disease occurrence maps.

**Table 2:** Terminology used to define the mode of transmission in this observation.

**Cases**	**Criteria**	**Remarks**
Imported cases	Individuals with a travel history to high-risk countries	Stage 1 transmission
Local cases	Close contact of imported casesClose contact of local cases	Stage 2 transmission (person to person)
Cluster	Two or more confirmed cases with epidemiological links based on disease characteristics and contact patterns (mass contact of local cases) (7)	Stage 3 transmission (community)

The guidelines from the Declaration of Helsinki (a set of ethical principles regarding human experimentation developed for the medical community by the World Medical Association) was followed during data collection from the respondents (9). The respondents were informed about the objective of this study, and their consents were acquired before the submission of the survey form.

### Data Analysis

(a) The Occurrence of COVID-19 in Bangladesh

We performed a descriptive analysis to present data on the cumulative confirmed cases, daily new cases, daily tests, cumulative death due to COVID-19, and age distribution of confirmed cases.

(b) Calculation of the Social Distancing Status of Different Districts
  - At first, we converted the status into numbers presented in Table 3.
  - Then, using this equation, we have calculated the ratings:
    - Rating = (total vote in “Very good” × 2) + (total vote in “Fairly good” × 1) + (total vote in “Good” × 0) + (total vote in “Too bad” × −1)
  - Average ratings = rating/total vote. The social distancing status was categorized based on the ratings obtained from the above calculations presented in Table 4.

**Table 3:** Social distancing status on a rating point (−1–+2).

**Status**	**Criteria of social distancing**	**Corresponding point**
Very good	Strictly maintained	2
Good	Avoided for several hours a day	1
Fairly good	Limited to aged and children	0
Too bad	Gathering of people hither and thither	−1

**Table 4:** Range on a rating point (−1–+2) used for social distancing status.

**Range**	**Status**
1.5–2.5	Very good
0.5–1.5	Good
−0.5–0.5	Fairly good
−1.5 to −0.5	Too bad

The map visualization was done by using the GeoJSON data from Geodash and OpenStreetMap®. The mapping was done with the Leaflet library in JavaScript.

(c) Predicting Cumulative Daily (Short-Term Forecast) Infected With Machine Learning

Our prediction analysis was based on the publicly available data of all and newly confirmed daily cases reported for Bangladesh from 8 March until 14 April 2020. We have used the Python model for finding a relationship between datapoints. Python has been the best open-source platform to use because of the less level of complexity, ready libraries, and easy deployment in a production environment (10). The x-axis represents the days of cases from the first day COVID-19 was reported, and the y-axis represents the number of confirmed cases.

(d) Predicting Future Trends (Long-Term Forecast) Using the SIR Model

We used the SIR model as a compartmental model that accounts for a number of susceptible individuals (S), a number of infected individuals (I), and a number of recovered or deceased (or immune) individuals (R). SIR model is a framework describing how the number of people in each group can change over time, and it allows us to describe the number of people in each compartment with the ordinary differential equation presented below:

$$\begin{array}{l} \frac{dS}{dt}=-\beta IS \end{array}$$

$$\begin{array}{l} \frac{dI}{dt}=\beta IS-\gamma I\\ \frac{dR}{dt}=\gamma I \end{array}$$

β is a parameter controlling how much the disease can be transmitted through exposure. It is determined by the chance of contact and the probability of disease transmission. γ is a parameter expressing how much the disease can be recovered in a specific period. Once patients are cured, they get immunity. There is no chance for them to go back susceptible again. When we can estimate the two values, there are several insights derived from it. If D is the average days to recover from infections, it is derived from γ.

$$\begin{array}{l} D=\frac{1}{\gamma } \end{array}$$

In addition, we can estimate the nature of the disease in terms of the power of infection.

$$\begin{array}{l} R_{0}=\frac{\beta }{\gamma } \end{array}$$

*R*_◦_ is the average number of new infections created by an infected individual when every person with whom they have contact is susceptible. *R*_◦_ > 1 means that each individual infects more than one other individual, and so the epidemic will grow exponentially. It is not directly associated with the number of recoveries, rather the average number of recoveries per unit time (i.e., the recovery rate). We do not know the values for the parameters β and γ yet, but we can estimate them, and then adjust them as necessary to fit the excess death.

The base class of the simulation is adopted from https://github.com/Lewuathe/COVID19-SIR with some modification to obtain *R*_◦_. The hypothesis remains: if the *R*_◦_-value is >1, the infection rate is greater than the recovery rate, and thus, the infection will grow throughout the population. If *R*_◦_ is <1, the infection will quickly decline since people are recovering faster than they are spreading the infection.

## Results

**Figure 1:**
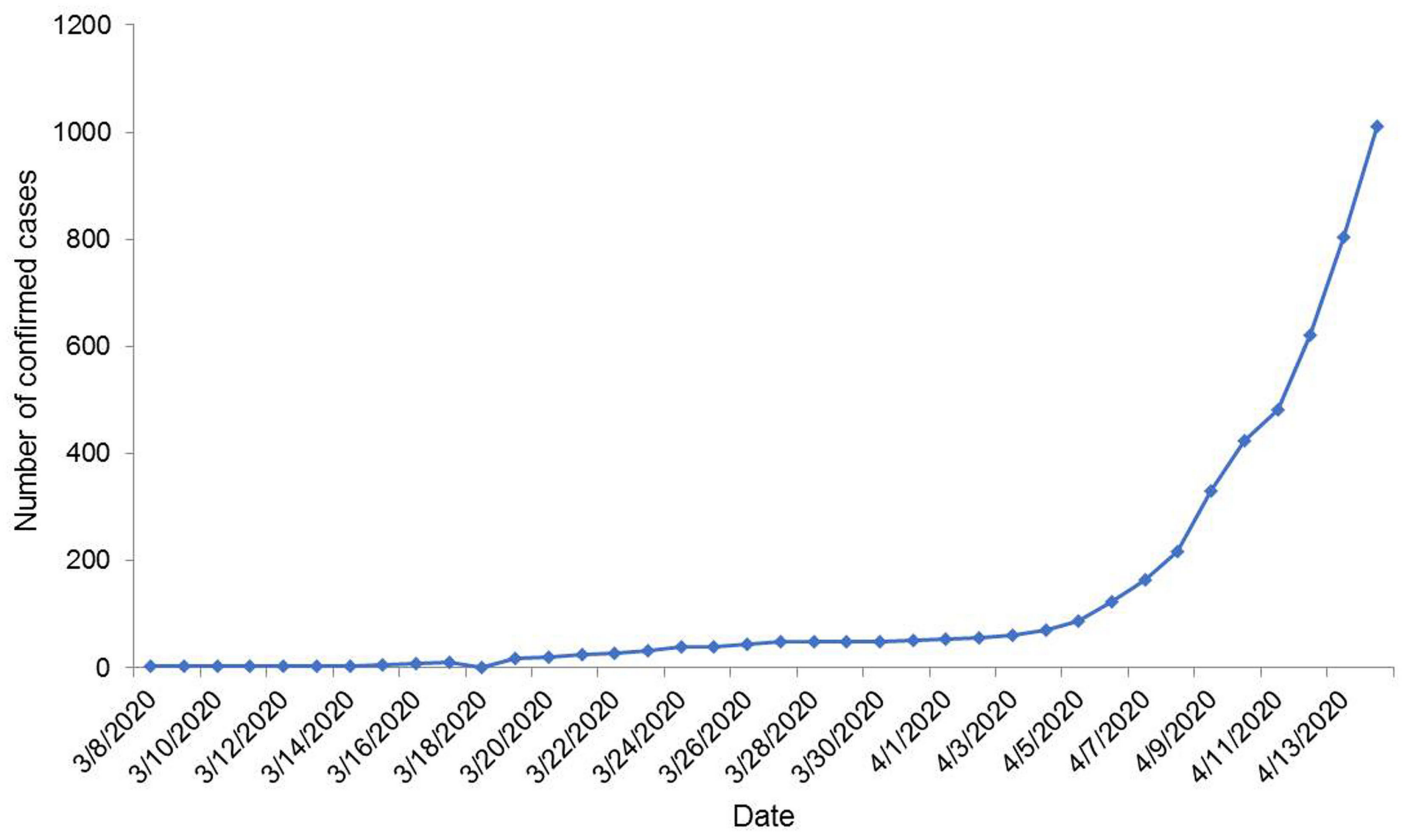
Temporal distribution of the number of COVID-19 confirmed cases in Bangladesh until 14 April 2020.

**Figure 2:**
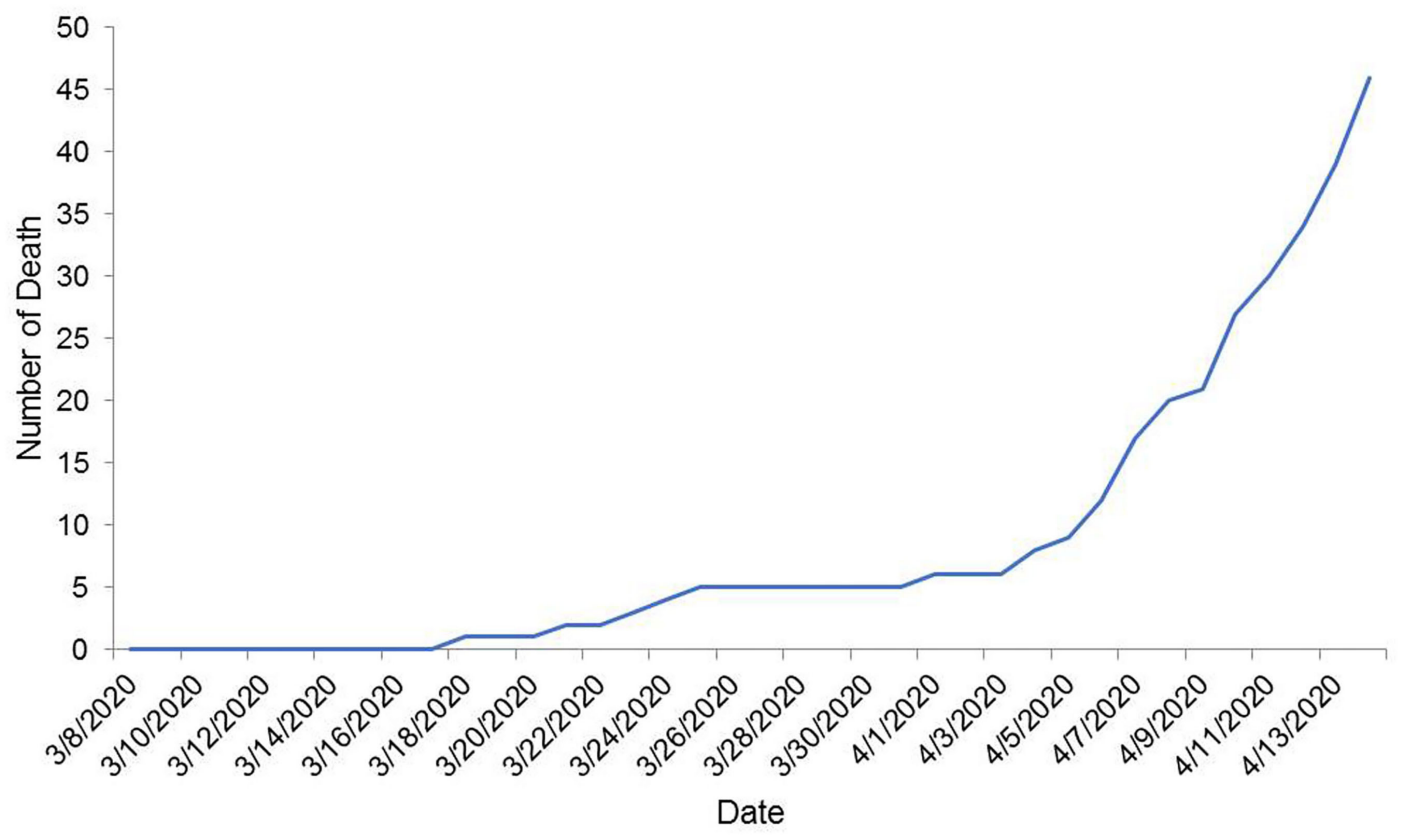
Temporal distribution of the number of cumulative death due to COVID-19 in Bangladesh up to 14 April 2020.

The number of confirmed COVID-19 cases and the temporal pattern of death due to COVID-19 are shown in Figures 1, 2, respectively. It roughly seemed that the cumulative number of positive cases was increasing exponentially, meaning community transmission at a later stage, although it had a flat state at an earlier stage. The pattern of death due to COVID-19 was roughly increasing starting from zero. After climbing up a bit, the curve stayed in plateau, as no death record was found for a few days. But after that, the cumulative trend continued to climb.

**Figure 3:**
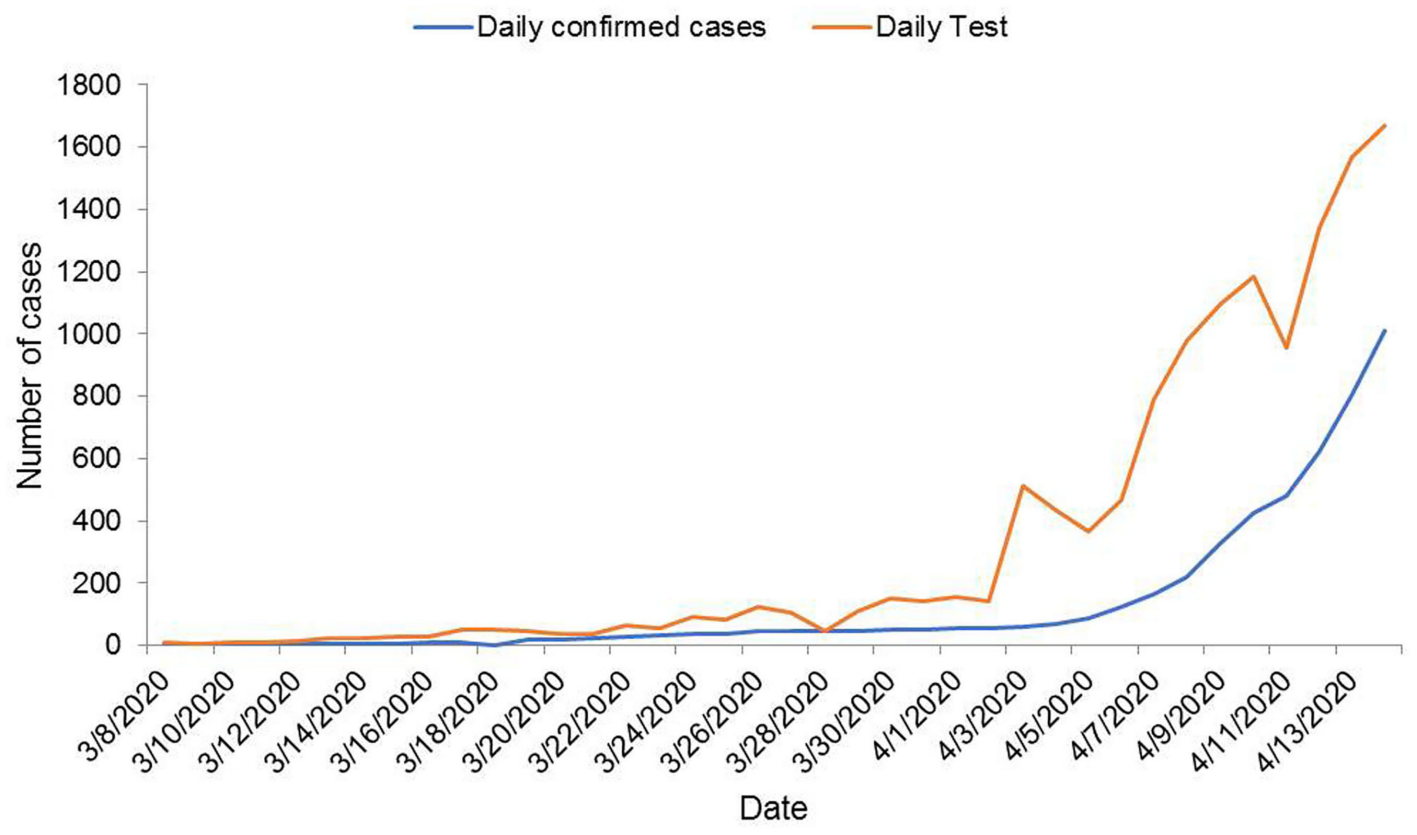
A plot of new confirmed COVID-19 cases vs. tests performed on a daily basis in Bangladesh until 14 April 2020.

**Figure 4:**
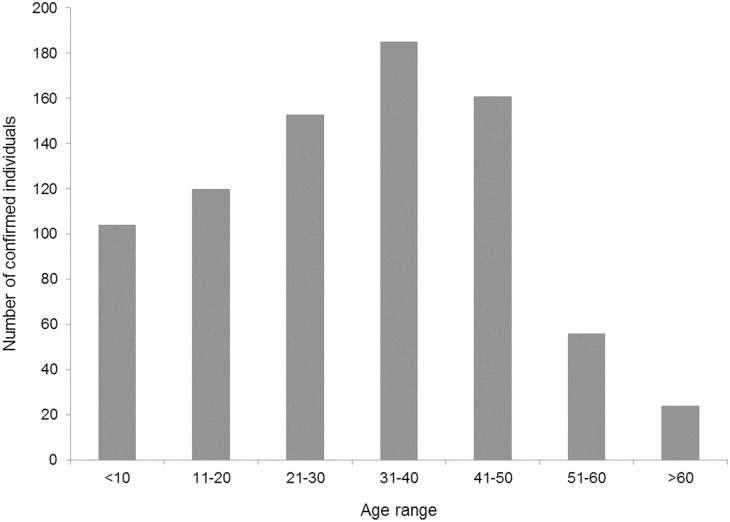
Distribution on the basis of age of all COVID-19 confirmed cases up to 14 April 2020.

Figure 3 compares the number of tests done daily and the number of COVID-19 positive cases each day. It shows that with the increase in the number of tests, the number of new positive cases also amplified. The age distribution of confirmed cases is presented in Figure 4. Considering the age range of the confirmed population, we observed the highest (23.03%) occurrence in the 31–40 years aged group, followed by 41–50 years (19.63%) and 21–30 years (18.52%) aged groups.

### Distribution of COVID-19 Cases and Social Distancing Status in Bangladesh

**Figure 5:**
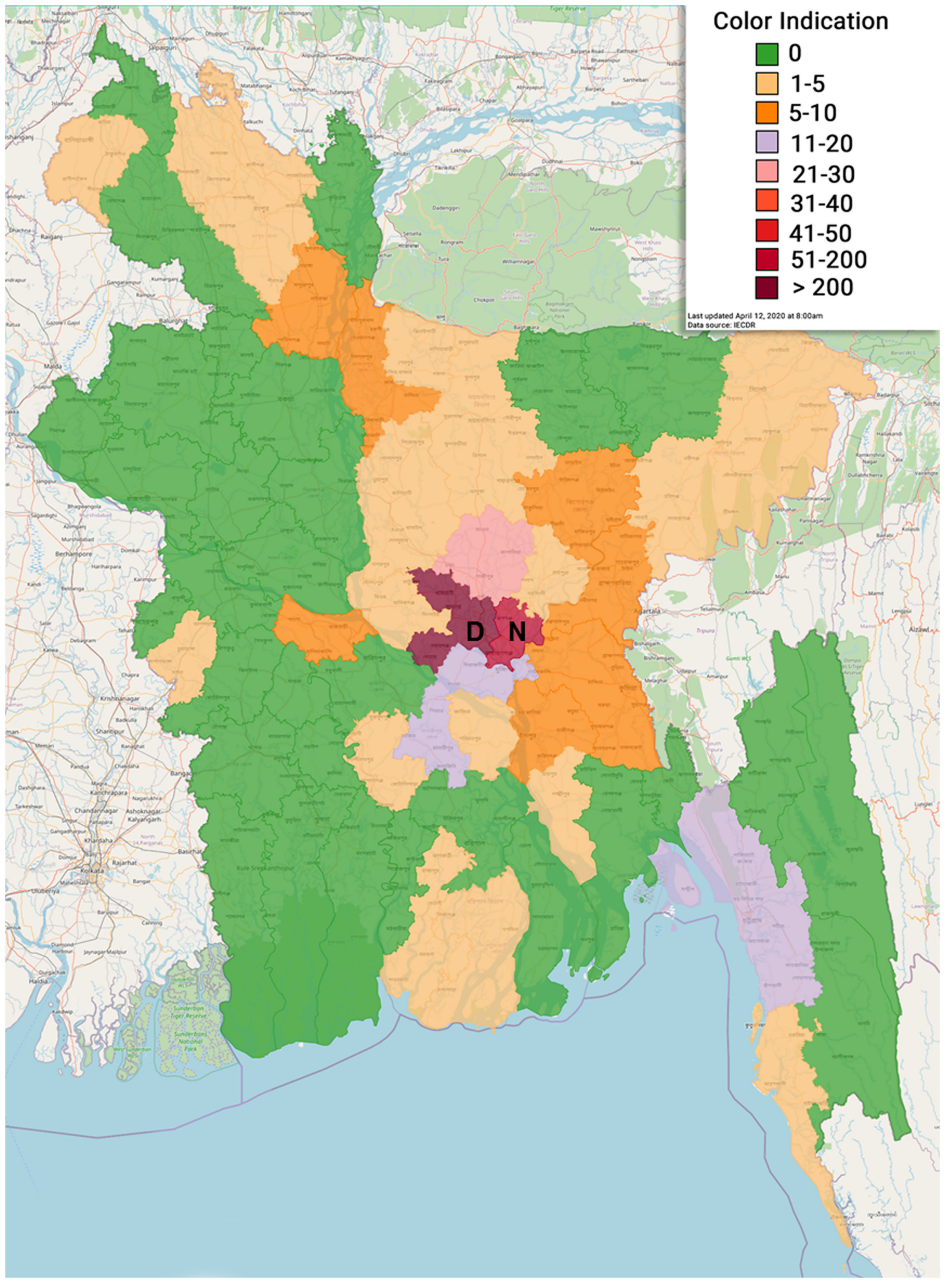
Geographical distribution of COVID-19 positive cases in various administrative districts of Bangladesh (D, Dhaka; N, Narayangonj).

**Figure 6:**
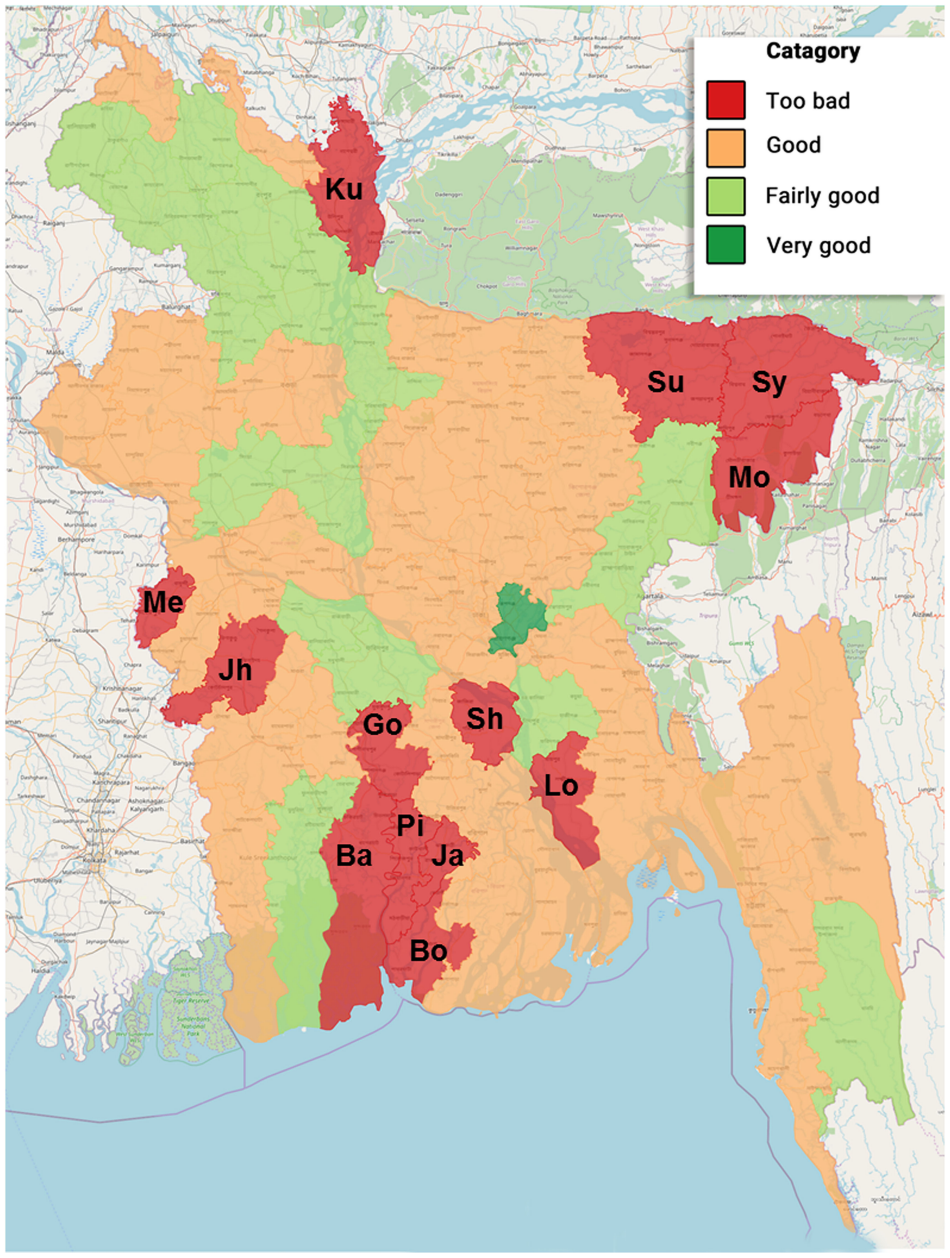
Scenario of social distancing situation in different administrative districts of Bangladesh during 8–14 April 2020 (Ba, Bagerhat; Bo, Borguna; Go, Gopalgonj; Ja, Jhalokati; Jh, Jhenaidah; Ku, Kurigram; Lo, Lakshmipur; Me, Meherpur; Mo, Moulvibazar; Pi, Pirojpur; Sh, Shariatpur; Su, Sunamgonj; Sy, Sylhet).

Maps (Figures 5, 6) show the distribution with the frequency of COVID-19-positive cases and give a brief idea on the situation of social distancing among people in Bangladesh. In addition, Table 5 presents the cluster areas, where community transmission was identified until the reporting time. It was observed that the highest cases were recorded in Dhaka, followed by Narayangonj, and these areas were at greater risks. It also indicated that the southwestern and southeastern zones were comparatively in better position, whereas the central, some part of northern, and central zones were comparatively in a risky position (Figure 5). Considering the social distance status, we found that the maximum area of Bangladesh covered fairly good to good and very good. Among the 64 districts of Bangladesh, five districts (Bagerhat, Borguna, Pirojpur, Shariatpur, and Lakshmipur) of the southern zone, one district (Kurigram) of the northern zone, two districts (Meherpur and Jhenaidah) of the eastern zone, and three districts (Sunamgonj, Sylhet, and Moulvibazar) of the western zone had poor quarantine situation (Figure 6).

**Table 5:** Characteristics of large clusters in Bangladesh as of 13 April 2020.

**Cluster location**	**Cluster size**	**Reporting date for the first case linked to cluster**	**Potential source of infection for first case**	**Leading hot zone by cluster with cause**
Dhaka city	313	18 March	Local case (close contact of imported cases–family member of Italian returnee)	Mymensingh; migration of working people.
Narayanganj	107	8 March	Imported case (Italian returnee)	Narshindhi, Chattagram; migration of working people
Gazipur	23	10 April	Close contact of local case (mass flow of garments and factory workers)	Mymensingh; migration of garments worker
Madaripur	11	8 March	Local case (close contact of imported cases–family member of Italian returnee)	
Gaibandha	5	22 March	Imported case (American returnee)	Rangpur; free movement within areas

**Figure 7:**
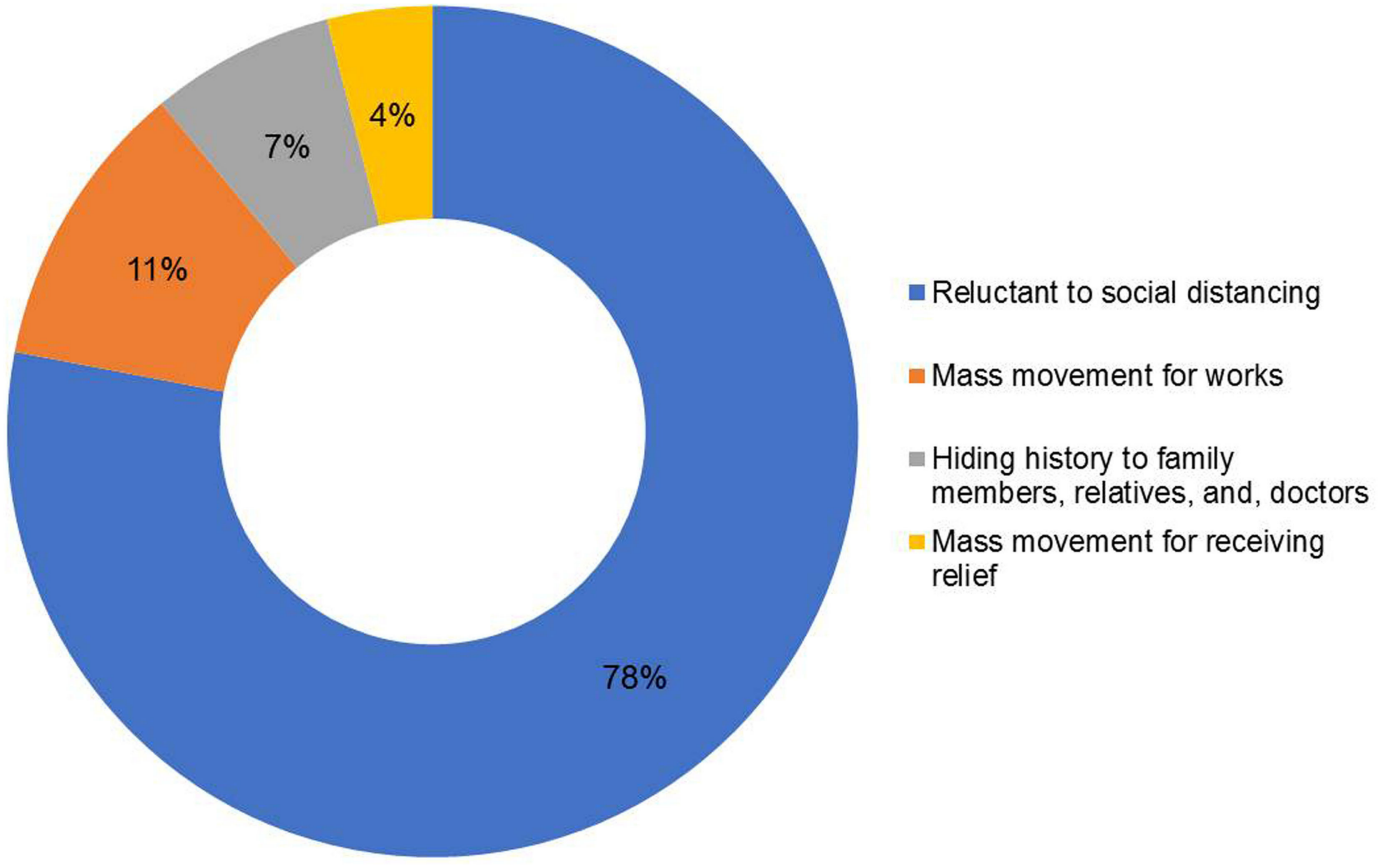
Factors facilitating the transmission of COVID-19 in Bangladesh on the basis of public opinion.

We found four risk factors facilitating the transmission of COVID-19 in Bangladesh. The result was formulated from public opinion and is shown in Figure 7. Improper quarantine or reluctance to social distancing was the vital factor that might influence the infection rate.

### Prediction for Future COVID-19 in Bangladesh

#### Short-Term Forecast

**Figure 8:**
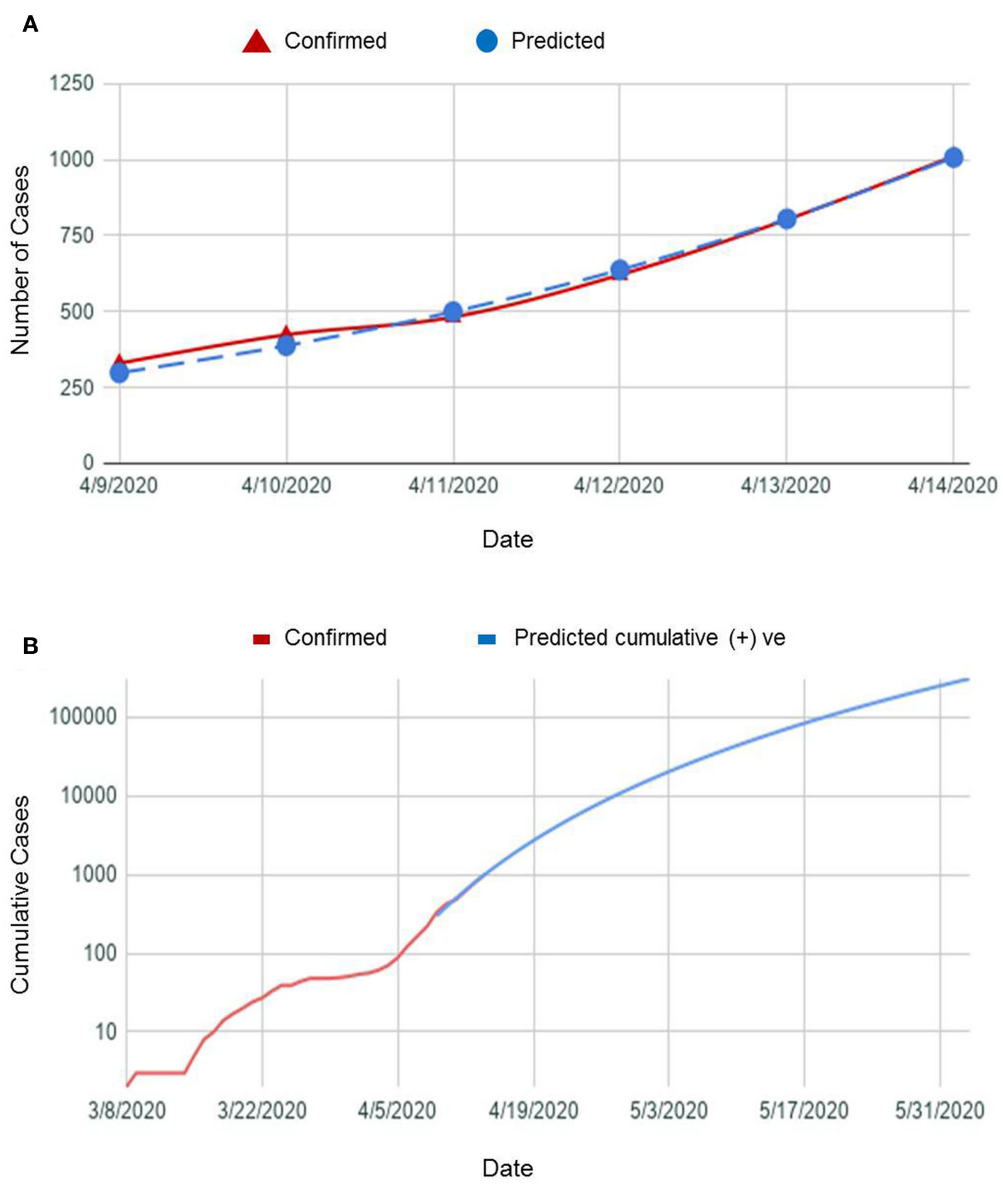
**(A)** A plot of COVID-19 confirmed cases vs. predicted cases in a week and **(B)** a graph showing predicted cumulative COVID-19-positive cases in near future.

We showed a 1-week prediction curve from 9 April (when the first curve from previous secondary data was prepared) to 14 April to justify the prediction level and found that the prediction curve and the actual curve completely fitted to each other. This prediction was done by assuming exponential growth with a single growth rate, i.e., on the logarithm scale, the trend of the graph was constant. This means that this prediction did not include directly any factor that might influence the curve. Rather, it can be assumed that all the conditions and factors were the same along time. Figure 8A shows that the trend was almost linear, and the prediction curve progressed straight forward. It showed that, on 9 April, the number of cumulative positive cases was around 300, and the number of predicted cumulative positive cases was very close to it. Gradually, the predicted curve followed the actual curve and coincided on 14 April. On the other hand, Figure 8B shows the number of predicted cumulative COVID-19-positive cases in the near future if all other conditions were unchanged.

It is clearly seen from the graph that if the other conditions were unchanged,

- by the end of April, the number will reach more than 10,000,
- by the mid-May, the number will reach more than 60,000, and
- by the end of May, the number will reach more than 250,000.

#### Long-Term Forecast

**Figure 9:**
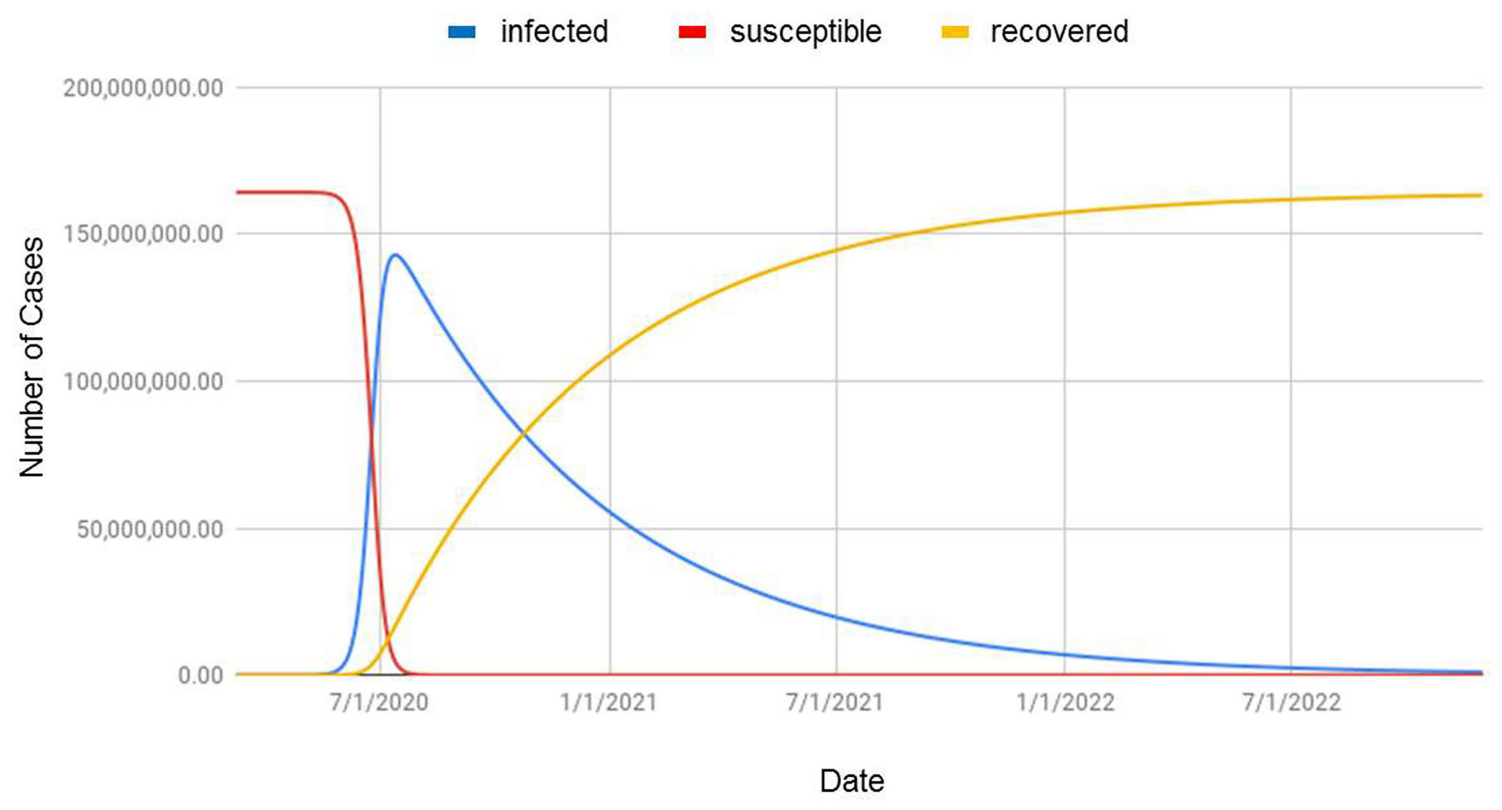
Prediction of COVID-19 situation based on the SIR model applied to populations in Bangladesh (infected = confirmed cases; susceptible = population at risk; recovered = cumulative cured or dead COVID-19 patients).

We used SIR mathematical and compartmental model for predicting the time when the number of infected cases would be reasonably close to the upper limit (when the pandemic will end). We obtained β = 0.187, γ = 0.006, *R*_◦_ = 6.924. *R*_◦_-value >1 indicates the infection rate is greater than the recovery rate, and thus, the infection will spread throughout the population. If the situation remained unchanged in Bangladesh, our finding revealed that we were likely to hit 150,209,981 cases in the future. The pandemic would end by December 2022 (Figure 9).

## Discussion

Epidemiological predicted data can be used to gain an understanding of population-level disease progression, which in turn is used to aid in decision-making and allocation of resources (11). Amid a critical and emergent situation related to extreme economic repercussions, we used mathematical modeling like linear regression and SIR to gather a numerical demonstration on the fate of COVID-19 in Bangladesh. This is the first study to report the transmission potential of COVID-19 in Bangladesh based on the mathematical prediction.

We presented the number of COVID-19 confirmed cases, the number of tests done, and the pattern of newly tested COVID-19-positive cases with respect to time. Initially, only a few tests were done. Only the suspected patients having a history of coming from abroad or people in contact with them were tested. Even if the patients had symptoms, they were not tested unless they met those conditions. The reason for this might be due to the limited testing facilities. However, gradually, people dispersed to various districts from the capital, and community transmission of the virus started. Simultaneously, testing facilities were increased in different places across the country.

During this observation, we found a similar increased pattern in new tests and new confirmed cases. From this finding, it can be assumed that if there were more tests, there might have been a greater possibility of having more positive cases. After the first confirmation of COVID-19, the number of daily new positive cases stayed within one digit and could be assumed as localized transmission only. Then, positive cases were increasing exponentially. The trend started to climb up, which indicated community transmission. Ma et al. (12) state that most pandemics follow an exponential curve during the initial spread and eventually flatten out. If so, it might be possible that there have already been a greater number of positive cases unidentified who would be the potential sources of community transmission in the future. Moreover, the increased number of death suggested observation of patient characters, which could not be presented due to the unavailability of data. Age distribution revealed that 62% confirmed cases were within 20–50 years age group. WHO (13) reported about 32 and 34% confirmed cases of the same age group in the USA and Spain, respectively. Unawareness and reluctance to social distancing and wandering here and there might be the cause for the occurrence of COVID-19 in this age group.

More positive cases were present in Dhaka city followed by Narayangonj during this study. Dhaka is the capital, having higher population density; people coming from other countries first landed here. There were more workers who were unaware of the critical situation, who kept working in factories or industries up to late March. This situation of Dhaka might be the reason for having the highest number of confirmed cases. Our survey on quarantine status revealed that the southwestern and southeastern zones were comparatively in better position, whereas the central, northern, and eastern zones were comparatively at risk. It is worthy to mention that the areas where a few (0–5) cases were identified had the too bad social distancing status. Unawareness of the people in these areas might be the cause behind poor social distancing status. However, these data would not be stable, as the quarantine situation changed over time. Thus, it would not be scientifically sound to predict future trends based on preventive measures, like quarantine, without real-time constant updating of data.

We identified four risk factors while considering the mode of transmission of COVID-19, such as reluctance to social distancing, a mass movement for work, a mass movement for receiving relief, and hiding the history of infection to family members, relatives, and especially doctors. It is difficult for people aged >60 years to go beyond the local or traditional myth and to understand the importance of isolation and quarantine. Up to the date of data collection, the quarantine situation seemed to be working smoothly in some places. However, it might not represent the whole or the actual scenario. If quarantine is not properly maintained, community spread would occur and the curve would upsurge exponentially. Several media reports confirmed that people coming from the cluster or hot zone tested positive, and they infected their surroundings, too. Sick individuals dispersed illegally from Narayanganj and Dhaka might have infected many people from places where no positive cases were reported earlier.

There were a few incidents attempting to escape isolation wards in hospitals specialized for COVID-19 patients and hiding overseas travel history. Many people with exposure to suspected cases of COVID-19 and some infected persons attempted to avoid the obligatory domestic isolation. A higher percentage of young people (age range, 20–50 years) was infected with COVID-19 due to this reluctance. Sometimes, suspected people hide history due to fear of isolation. Since lockdown was ongoing for a prolonged period, underprivileged people come out to take relief or to search for livelihood. Moreover, the migration of laborers and workers worsened the situation. Recently, there were several cases of potential COVID-19-positive individuals having clear symptoms who hid the history to physicians while taking treatment. Later, those physicians tested positive, and some hospitals had to be partially or completely shut down because of this. It might be another source of infection, as they (medical personnel) treated other susceptible patients.

Individual health well-being decision is guided by social standards, peer's suggestion, and media influence. Considering the transmission mode of an infectious disease in such a social environment, various mathematical models are incorporated to influence the individual's perceptions in preventing the spread of infection (14). That is why we focused on the SIR model, which has better abilities for long-term forecasts, to predict the fate of a pandemic infection like COVID-19 in Bangladesh. We obtained *R*_◦_ = 6.924. Ridenhour et al. (15) pointed *R*_◦_ as a complicated property of an epidemic, which is model specific, and depends on various factors like the population being studied, the host, the pathogen, and often the specific strain of the pathogen. Therefore, this number may vary geographically due to changes in the environment, population structure, viral evolution, and immunity as well as healthcare and immigration policies.

Our SIR model revealed that we might have 150,209,981 cases in the future if all the factors were unchanged, and the pandemic would end by December 2022. Although this simulation is just an assumption, the predicted number of cases is extremely high and would ~86.52% of the total population in the country in 2022 (16). The reasons behind such a massive number of COVID-19 patients might be due to ignorance and unwillingness of the people about personal hygiene and social distancing, a high population density, unavailability of basic medical equipment and facilities in rural areas, and lack of effective treatment strategies (4). To prevent a dire situation, our goal must be directed to decrease β and increase γ. To decrease β, some measures need to be taken like effective isolation and quarantine measures, wearing a mask in public places, washing hands regularly, and limiting social interaction. Social distancing must be implied strictly, as it can reduce the epidemic by up to 62% (17). To increase γ, effective treatment is essential in obtaining recovery from COVID-19. These trajectories made by SIR could serve as a means for governments, businesses, and individuals to plan and mitigate for such a spike in infected cases by giving priorities on personal hygiene and control measures and refraining from mass gatherings. The Government of Bangladesh barred mass travel via water, rail, and domestic air routes and public transports as well as mass gathering after experiencing six official deaths of COVID-19-positive patients. Armed forces were deployed to tackle mass gatherings. The onward trend of the COVID-19 outbreak in China declined significantly within 50 days due to the emergency response plan and public health efforts (18, 19). The Prime Minister extended nationwide lockdown until May 5 to maintain the home quarantine apart from emergency services, e.g., hospitals, drug dispensaries, and media outlets open, which resulted in the decline of the total number of identified cases (7,667 positive cases reported by IEDCR on 30 April 2020), although our prediction was 10,000 positive cases by the end of April 2020.

Because of real-time changes in daily data, the prediction will accordingly change. Hence, the results from this paper could be used only for qualitative understanding and a reasonable estimate of the nature of the outbreak. These models expect all the seed cases to be symptomatic, which may differ from the real numbers because of an uncertain number of asymptomatic people. We need to ascertain a proper model and assumption using real data, impose strict lockdown measures, and isolate all the possible carriers of COVID-19. The predictions were made without considering the dynamic factors that potentially might affect the curve due to the lack of proper data. In addition, these estimations could alter significantly if there were any large takeoffs within the drift. None of the model transmission or community transmission enters in all districts of Bangladesh until 14 April 2020. This study provides an overview and gross assumption of the future situation in Bangladesh. Furthermore, this study highlighted the importance of rapid case identification and subsequent isolation, the practice of personal hygiene, and strict maintenance of social distancing as control measures to reduce the onward chains of COVID-19 transmission.

## Data Availability Statement

The raw data supporting the conclusions of this article will be made available by the authors, without undue reservation.

## Ethics Statement

Ethical review and approval was not required for the study on human participants in accordance with the local legislation and institutional requirements. Written informed consent for participation was not required for this study in accordance with the national legislation and the institutional requirements.

## Author Contributions

RH, ASD, JKM, THK, AAN, MSS, and NSJ contributed to study design. RH, ASD, JKM, THK, AAN, and MSS contributed to data collection, compilation, and data analysis. MH and NSJ contributed to the literature search and critical review. ASD, JKM, MH, and NSJ contributed to the design of tables and figures. All authors contributed to the article and approved the submitted version.

## Conflict of Interest

The authors declare that the research was conducted in the absence of any commercial or financial relationships that could be construed as a potential conflict of interest.

## Supplementary Material

Supplementary Material S1 Coding language of SIR model.
