# Supplementary figures and images for: Prediction of Epidemics Trend of COVID-19 in Bangladesh
Source: Front Public Health. 2020 Nov 30;8:559437. doi: 10.3389/fpubh.2020.559437 (PMC7734053; doi:10.3389/fpubh.2020.559437)

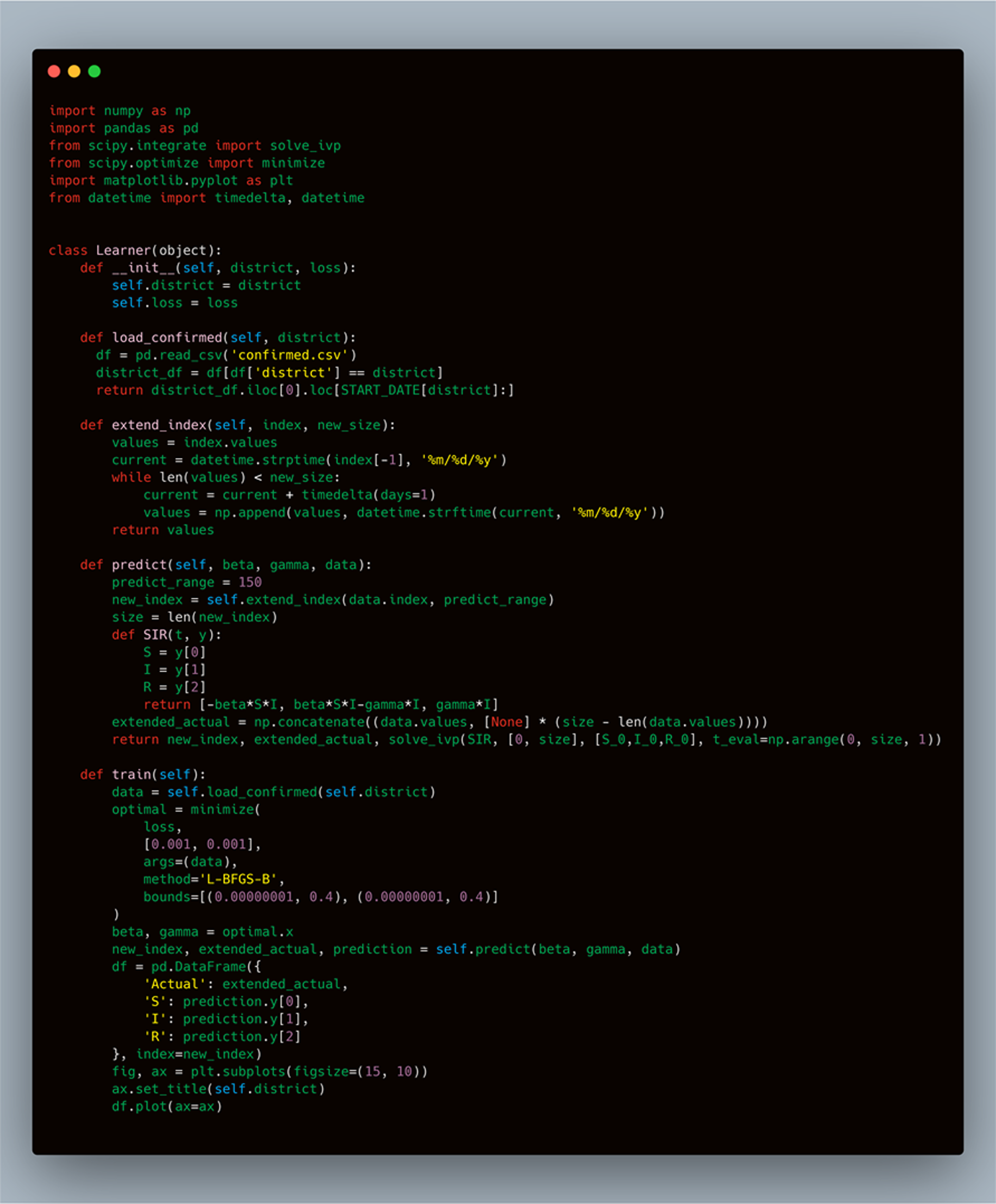

Supplement: Supplementary Material S1 — Coding language of SIR model. [file Image_1.TIF]
